# Toward Perceived Sustainable Employability: Capabilities of Secondary School Teachers in a South African Context

**DOI:** 10.3389/fpsyg.2022.842045

**Published:** 2022-05-04

**Authors:** Tessa de Wet, Sebastiaan Rothmann

**Affiliations:** Optentia Research Unit, North-West University, Vanderbijlpark, South Africa

**Keywords:** capabilities, sustainable employability, flourishing at work, organizational citizenship behavior, intention to leave, South Africa, secondary school teachers

## Abstract

This study aimed to identify the capabilities of secondary school teachers – valued aspects of work that are enabled and can be realized – and investigate the effects of these capabilities on three functionings: flourishing at work, organizational citizenship behavior, and intention to leave. A convenience sample of secondary school teachers (*N* = 144) in the Gauteng province in South Africa participated in the study. The teachers responded to the Capability Set for Work Questionnaire, Flourishing-at-Work Scale – Short Form, Organizational Citizenship Behavior Questionnaire, and Intention to Leave Scale. The results showed that three capabilities were most likely to form part of the capability set of teachers: using knowledge and skills, building and maintaining meaningful relationships at work, and contributing to something valuable. Capabilities least likely to form part of the capability set included having a good income, involvement in important decisions, and developing knowledge and skills. The capability set was a strong predictor of emotional, psychological, and social well-being, and a moderate predictor of organizational citizenship behavior and intention to leave. A capability set for work, rather than single work capabilities, seemed to be critical for the sustainable employability of secondary school teachers.

## Introduction

Teacher well-being, performance, and retention are critical research themes in occupational health psychology (OHP). Recently, the OHP literature shifted toward enabling workers to see themselves as part of building flourishing societies and lives (Van der Klink et al., 2016; Van der Klink, 2019). This shift recognizes how social and working conditions can enable individuals and institutions to flourish, which is necessary for maintaining sustainable employability (Van der Klink, 2019). If individuals accept work as an important part of their lives, it must not be viewed as a burden (Abma et al., 2016). Taking on this challenge requires new attention to the interconnection between work and well-being by focusing on the capabilities of teachers and their sustainable employability. Therefore, from a capability approach perspective, employee well-being, performance, and retention refer to the extent to which individuals can be and do what they have rationale to value (Sen, 1999) in order to flourish, perform, and be intent on staying in a job (Keyes, 2002).

Various studies of teacher well-being, performance, and retention have been conducted in sub-Saharan Africa (De Wet, 2022). Jackson et al. (2006) examined the effects of job demands and resources on secondary school teachers’ burnout and work engagement. Janik and Rothmann (2015) and Fouché et al. (2017) studied the relationship between meaningful work and secondary school teachers’ intention to leave. Furthermore, Janik and Rothmann (2016) investigated the effects of the relational context of secondary school teachers on their work engagement. However, these studies utilized the job demands-resources model as framework (Demerouti et al., 2001). No comprehensive studies have been conducted regarding teacher capabilities and their associations with the well-being, performance, and retention of secondary school teachers in sub-Saharan Africa.

## Literature Overview

### The Capability Approach

Sen (1985a,b) conceptualizes the CA as an approach that encapsulates going beyond resources and utility (as in traditional economic approaches) to include individuals’ capabilities, i.e., their freedom and opportunities to achieve valued outcomes (Nussbaum, 2011; Alkire and Santos, 2014). Sen (1992) believes that equity of capabilities is more important than equal means or resources. Furthermore, Claassen (2018) points out that the conversion of resources into capabilities must consider human diversity.

Sen’s (1985a,b,^1992^) CA is the basis for Van der Klink et al.’s (2016) model of sustainable employability. The latter model suggests that teachers’ capability set contributes to their sustainable employability (Abma et al., 2016). Sustainable employability means that workers are provided with conditions that allow them to be safe and healthy, and fulfilling work environments to benefit them and their organizations (Abma et al., 2016; Van der Klink et al., 2016).

Sen (2008) is reluctant to present a list of capabilities that should form part of a person’s capability set (Claassen, 2018). Sen (2008) maintains that individual values need public scrutiny because adaptive preferences can form in unfavorable circumstances. Consequently, Sen (2008) endorses a proceduralist approach (Claassen, 2018), focusing on objective rather than subjective evaluations (DeHaan et al., 2016). Although the CA emphasizes the need for value assessment when assessing people’s lives, implementation has often been difficult (Hirai, 2021). The CA may accurately outline important social and material conditions and affordances objectively, but is not necessarily accurate in reflecting on people’s lives based on subjective values. Therefore, in contrast to Sen’s (2008) approach, Nussbaum (2011) identifies 10 human capabilities essential to people’s flourishing. She argues that a capability list should be the guiding principle for securing individual values. Accordingly, Hirai (2021) points out that it is possible to ask people about the extent to which they have attained their values and the importance of each value. Indeed, studies have operationalized capabilities and shown empirically that subjective well-being is related to capabilities (Anand et al., 2009; DeHaan et al., 2016). The current study combined the approaches of Sen (1985a,b) and Nussbaum (2011) to explore the capabilities and functionings of secondary school teachers.

Van der Klink (2019) argues that people should see work from the perspective of personal and social values instead of mere production. Unfortunately, practitioners and policymakers often regard work as a factor of production. Hence, services aiming for participation and well-being should not be sold as if they will always add to people’s efficiency and productivity (i.e., as if the purpose is only to make money). Moreover, models of work-related well-being such as the Michigan model (French and Caplan, 1972) and the job demands-resources (JD-R) model (Demerouti et al., 2001) do not adequately account for the crucial role of values and context in individuals’ work abilities. Instead, a value case should be made for such services, which should aim to contribute to individuals’ health and well-being. Many people choose jobs based on their preferences, about which they feel good, and which interest them. When individuals engage in work that they value, they should flourish.

The CA can be used to identify important work-related values and examine how people are enabled and are able to achieve these at work (Van der Klink et al., 2016). Sen’s (1985a,b) approach to capability gave rise to the concept of valuable work. According to Van der Klink et al. (2016), human beings are increasingly looking for valuable work or work that fulfils their values. Van Casteren et al. (2021) see meaningful work (indicated by the value assigned to specific work) as critical in creating health, well-being, and sustainability in the employment relationship. A framework presented by Gheaus and Herzog (2016) enables consideration of “the goods of work” that people value: (a) salary and wages; (b) attaining excellence; (c) making a social contribution; (d) experiencing community; and (e) gaining social recognition.

Work values become capabilities when employees find them important, contextual factors enable them to achieve such values, and they do so themselves (Abma et al., 2016; Van der Klink, 2019). Full capability can be obtained when workers value the capabilities provided, have enough opportunities and freedom to engage with the capability aspect, and succeed in achieving outcomes. Employees can draw on personal, social, and environmental resources in the employment relationship. The opportunity and freedom to achieve outcomes in their (working) lives distinguish the CA as a framework for studying well-being at work and the conditions leading to sustainable employability.

The CA identifies a capability set as a bundle of options available to facilitate a range of beings and activities that people value (Sen, 1992). Research (Abma et al., 2016) has indicated a generic capability set for work. Individuals and society are jointly responsible for building and sustaining the necessary capabilities for a valuable, flourishing lifestyle (Van der Klink et al., 2016). The capability set includes seven capabilities: using and developing knowledge and skills, setting own goals, being involved in important decisions, building meaningful relationships at work, maintaining these, contributing to something valuable, and having a good income. Abma et al. (2016) identified the capabilities from a qualitative study (based on employee interviews), a literature review, and expert meetings. There is alignment between these capabilities and other frameworks available in the social sciences literature (e.g., Arendt, 1958; Jahoda, 1982; Gheaus and Herzog, 2016).

### Functionings of Secondary School Teachers

According to Sen (2008), capabilities reflect the freedom to achieve valuable functionings. In the CA, functionings are the achievement of valuable beings and doings (Sen, 1985a). Abma et al. (2016) researched the capabilities and functioning of employees in the Netherlands, focusing on work outcomes, including work functioning, work performance, work ability, sickness absence, and hours worked. They found significant correlations between capabilities and work outcomes.

Recent developments in positive psychology demonstrate growing interest in human flourishing. Humans need to express and realize their abilities and capabilities (Ryan et al., 2013; DeHaan et al., 2016). The CA provides insight into the social and material conditions necessary to exercise human abilities. A person’s ability to engage in activities he/she values is considered an indicator of his/her well-being (Sen, 2008). The perceived quality of individuals’ work-lives affects their overall well-being (Rath and Harter, 2014). This is referred to as subjective well-being – an individual’s perception of feeling good and functioning well – which affects employees’ work-life and productivity (Harter et al., 2003).

Concerning functionings, studies have shown that a chain consisting of elevated job demands, burnout, and ill-health can lead to powerlessness or refusal of teachers to perform and that job resources can lead to work-related well-being and organizational commitment (Jackson et al., 2006; Fouché et al., 2017). Therefore, the envisaged outcome of the CA-based sustainable employability model – perceived individual well-being in the form of work-life quality – can assist in garnering a greater understanding of how individuals function on a scale from flourishing to languishing at work. In addition to individual well-being, outcomes such as performance and intention to leave are critical.

Rothmann (2013) suggested a model for *flourishing at work*, defined as working within the ideal range of employees’ functionality in an organizational context, which includes feeling good (emotional well-being), functioning well (psychological well-being), and fitting in (social well-being). This model provides a multidimensional way to understand and develop optimal employee functioning in occupational contexts. Firstly, emotional well-being consists of job satisfaction and positive affect. Secondly, psychological well-being includes satisfying the psychological needs for autonomy, competence and relatedness, engagement, learning, meaning, and purpose. Thirdly, social well-being includes social acceptance, social actualization, social contribution, social coherence, and social integration (Rothmann, 2013; Redelinghuys et al., 2019). Studies have indicated that experiences of work affect people’s flourishing and that work role fit, job characteristics, co-worker relationships, and remuneration positively affect individuals’ flourishing (Rothmann, 2014).

Borman and Motowidlo (1997) distinguish two types of performance: contextual and task performance. Task performance refers to activities that turn materials into goods or services produced by the organization or enhance the organization’s ability to function efficiently as a whole (Borman and Motowidlo, 1997). Employee contextual performance refers to how well employees engage in activities to shape the necessary contexts for task activities to succeed. Abma et al. (2016) showed that capabilities were statistically significantly related to task performance. However, the current study focused on the association between capabilities and contextual performance. *Organizational citizenship behavior* (OCB; Smith et al., 1983) is a type of contextual performance. It refers to actions taken to help others in the organization or demonstrate conscientiousness in support of it. Factor analyses of item responses suggest two types of behavior: (a) altruism, or helping others, and (b) generalized compliance, defined as adhering to organizational rules and procedures (Organ, 1988; Möller and Rothmann, 2019).

Most organizations strive to retain employees because employee turnover can be detrimental to organizations and employees (Holtom et al., 2008). A person decides to leave a job before resigning. Therefore, employees’ *intention to leave* (ITL) is a good indicator of turnover (Coward et al., 1995; Mor Barak et al., 2001). In addition, intention to leave discloses an employee’s conscious frame of mind of being in favor of working elsewhere (Tett and Meyer, 1993). From 2011 to 2015, 29,741 South African educators left their profession (Liebenberg and Hattingh, 2017). From 2008 to 2013, 43.9% of educators resigned from their jobs. Liebenberg and Hattingh (2017) found that low morale and low teacher-principal relationships among educators were related to resignations. Relationships were found between flourishing at work and intention to leave the teaching profession (Redelinghuys et al., 2019).

### Current Study

Secondary school teacher functionality research in sub-Saharan Africa has focused on identifying the presence (or lack) of various resources that can affect teachers’ sustainable employability capabilities (Abma et al., 2016). Rothmann (2021) indicated that secondary school teachers were the second-highest risk group for burnout in South Africa. However, there is very little integrated knowledge of the sustainable employability of such educators in South Africa from the CA. Although some research has been conducted at primary school level (Buckler, 2012; Tao, 2013, 2014), research on secondary school teachers in South Africa is scarce. This study aimed to identify the work capabilities of secondary school teachers – valued aspects of work that were enabled and achieved – and investigate their effects on three well-being functionings: work flourishing (i.e., emotional, psychological, and social well-being), organizational citizenship behavior, and intention to leave.

The following hypotheses are proposed:

**Hypothesis 1:** Higher scores on each of the seven work capabilities are associated with emotional well-being (H1a), psychological well-being (H1b), social well-being (H1c), organizational citizenship behavior – altruism (H1d), organizational citizenship behavior – generalized compliance (H1e) and lower scores on intention to leave (H1f).

**Hypothesis 2:** Seven capabilities, namely using and developing knowledge and skills, setting own goals, being involved in important decisions, building meaningful relationships at work, maintaining these, contributing to something valuable, and having a good income, are associated with a capability set (H2a).

**Hypothesis 3:** A higher score on the capability set is associated with emotional well-being (H3a), psychological well-being (H3b), social well-being (H3c), organizational citizenship behavior – altruism (H3d), and organizational citizenship behavior – generalized compliance (H3e) and lower scores on intention to leave (H3f).

## Materials and Methods

### Participants

The study population comprised secondary school teachers from independent and public schools in three districts of Tshwane. These districts were identified as the area of study as it is situated in the smallest and most densely populated of all the South African provinces (Gauteng), making it possible to collect representative data. The Tshwane districts (North, South, and West) include diverse schools in terms of demographic characteristics (rural/urban and socio-economic status). Cost and practical considerations and the diversity in the three districts of Tshwane led to the decision to draw a probability sample in this South African context. The strategy was to sample at least 400 secondary school teachers in the three districts (with a minimum of 10 teachers per school). All schools in the districts that were contactable were contacted. Unfortunately, only 144 teachers responded to the survey. Due to the COVID-19 pandemic, the response rate was relatively low. In addition, strict ethics rules enforced by the ethics committee complicated the data gathering. However, it was still possible to obtain a reasonable sample of teachers. A total of 36 (17.9%) of the participants were employed in the Tshwane North district, 160 (79.6%) in Tshwane South, and five (2.5%) in Tshwane West. For those who indicated what language English was for them, 17 (16.7%) indicated English as their home language, 74 (72.5%) as their first additional language, and 10 (9.8%) as their second language. A total of 27 participants (26.5%) indicated that they were engaged in another job over and above teaching.

### Measuring Instruments

Four questionnaires were utilized in this study: the Capability Set for Work Questionnaire, Flourishing-at-Work Scale – Short Form, Organizational Citizenship Behavior Questionnaire, and Turnover Intention Scale.

The *Capability Set for Work Questionnaire* (CSWQ; Abma et al., 2016) measures employees’ capability sets. The CSWQ measures seven of the capabilities comprising the capability set: (a) use of knowledge and skills; (b) development of knowledge and skills; (c) involvement in important decisions; (d) building and maintaining meaningful contacts at work; (e) setting own goals; (f) having a good income; and (g) contributing to something valuable. For each capability, three questions are asked: “How important is it for you to … [capability]?”, “Does your current work offer you enough opportunity to … [capability]?”, and “To what extent do you succeed in … [capability]?” Responses are rated on a Likert scale from 1 (*not at all*) to 5 (*very much so*). Van Elteren (2016) found a Cronbach alpha coefficient of 0.91 for the CSWQ.

Concerning construct validity, Abma et al. (2016) found that employees’ capability sets were strongly related to work role functioning, work ability, work performance, and worked hours, while Van Gorp et al. (2018) found that the CSWQ related to health and work outcomes.

The *Flourishing-at-Work Scale – Short Form* (FAWS-SF; Rautenbach and Rothmann, 2017) comprises 19 items, rated on a scale from 1 (*never*) to 6 (*every day*). Participants must indicate how regularly they have experienced aspects at work during the preceding month. The scale contains three dimensions: emotional well-being (EWB), psychological well-being (PWB), and social well-being (SWB). EWB includes four questions about positive affect and job satisfaction (e.g., “During the past month at work, how often did you experience real enjoyment in your work?”). PWB includes questions about six dimensions: autonomy, competence and relatedness satisfaction, learning, meaningful work, and engagement (e.g., “During the past month at work, how often did you become enthusiastic about your job?”). SWB includes questions about five dimensions: social acceptance, actualization, coherence, contribution, and integration (e.g., “During the past month at work, how often did you feel you really belonged at this school?”). Acceptable reliabilities ranging from 0.92 for the full scale and 0.81 to 0.86 for the subscales of the FAWS-SF were found in studies of secondary school teachers in South Africa (Redelinghuys et al., 2019).

The *Organizational Citizenship Behavior Questionnaire* (OCBQ; Konovsky and Organ, 1996) was used to measure organizational citizenship behavior. The questionnaire consists of 10 items rated on a scale from 1 (*never*) to 6 (*every day*). Confirmatory factor analysis indicated that the instrument encompassed two dimensions: altruism and generalized compliance. The altruism component includes items such as “To what extent do you engage in the following behaviors at work: willingly give your time to help others who have work-related problems?” The generalized compliance component has items such as “To what extent do you engage in the following behaviors at work: take action to protect your secondary school from potential problems?” Möller and Rothmann (2019) found proof for the construct validity and internal consistency (ρ = 0.80 for altruism, ρ = 0.82 generalized compliance) of the OCBQ.

The *Turnover Intention Scale* (TIS; Sjöberg and Sverke, 2000) measures individuals’ intention to leave the job. Three items measure a single dimension: “I am actively looking for other jobs,” “I feel that I could leave this job,” and “If I were completely free to choose, I would leave this job.” These are rated on a five-point Likert scale from 1 (*strongly disagree*) to 5 (*strongly agree*). The instrument has been utilized and proven valid and has provided reliable results (Redelinghuys et al., 2019; Redelinghuys and Rothmann, 2020).

### Research Procedure

Before commencement of the study, scientific and ethical clearance was obtained from a reputable institution for higher education. Ethics approval was granted by the Health Research Ethics Committee (HREC) at North-West University (NWU-00430-19-A1). Permission for the study was also obtained from the research division of the Gauteng Department of Education (GDE). The data collection phase coincided with the start of the ongoing COVID-19 pandemic lockdown period in South Africa, necessitating remote communication and data gathering. Introductory materials were developed and made accessible electronically. The questionnaires were administered electronically or in paper format between June 2020 and July 2021. Due to COVID-19 physical proximity restrictions, school principals as gatekeepers were contacted telephonically and by email to introduce the study and request participation. Willing principals submitted a letter of goodwill and a school questionnaire. The survey questionnaire was made available to them for distribution to teaching staff. In addition, the principals mediated contact with secondary school teachers. Finally, the data were captured on Microsoft Excel.

### Data Analysis

Mplus 8.6 (Muthén and Muthén, 1998–2021) and SPSS27 (IBM Corp, 2020) were used to analyze the results. The measurement models of flourishing at work, intention to leave, and organizational citizenship behavior were tested using the weighted least square mean and variance adjusted (WLSMV) estimator. Various indices were used to assess model fit: chi-square (χ^2^), the standardized root mean square residual (SRMR), the root mean square error of approximation (RMSEA), the Tucker–Lewis index (TLI), and the comparative fit index (CFI). Lower values indicate better fit on all indices, except for the CFI and TLI, where higher values indicate better fit (Wang and Wang, 2020). Omega coefficients (McDonald, 1999) were computed to estimate the reliability of scales.

Following the procedure suggested by Abma et al. (2016), a summary score was calculated for each capability aspect to assess whether it formed part of the teacher’s capability set. A capability aspect was included in teachers’ capability set if they found the aspect important, were enabled to achieve it, and succeeded in achieving it. Teachers who found an aspect important (A), but lacked the opportunity to realize it (B) or failed to realize it (C) might demonstrate ineffective functioning. In contrast, teachers who regarded a capability aspect as important, were enabled to realize it, and succeeded in achieving it might function well. An aspect (range 1–5) was scored as part of the capability set when it was considered important (A = 4–5) and when the work environment offered sufficient opportunities (B = 4–5) and made it possible to realize it (C = 4–5). When teachers responded as follows, a capability aspect was not considered part of the set: (i) the capability was important (A = 4–5), but the workplace did not provide enough opportunities (B ≤ 3); (ii) the capability aspect was important (A = 4–5), but the person could not achieve it (C ≤ 3); or (iii) the workplace offered sufficient opportunities (B = 4–5), but the person could not achieve the aspect (C ≤ 3).

Descriptive statistics were computed to describe the data. Where applicable, Pearson correlations (*r*) or point-biserial correlations (*r*_*pb*_) were computed to assess the associations between variables (Field, 2016). Effect sizes (Cohen, 1988) were used to assess the correlations between variables, with the following cut-off values: *r* or *r*_*pb*_ > 0.50 (large effect), *r* or *r*_*pb*_ > 0.30 (medium effect), and *r* or *r*_*pb*_ > 0.10 (small effect). Finally, standard multiple regression analyses were used to investigate the effects of demographical variables and capabilities on emotional, psychological, and social well-being, organizational citizenship behavior, and intention to leave.

## Results

### Descriptive Statistics of the Capabilities

Participants rated each capability on three dimensions: value, opportunity, and achievement. Table 1 indicates that, for work values, the following percentages of secondary school teachers reported placing a high value on the different capability components: use of knowledge and skills (97.2%), contributing to something valuable (92.9%), development of knowledge and skills (91.5%), building and maintaining meaningful relationships at work (91.4%), setting own goals (90.1%), having a good income (88.7%), and involvement in important decisions (82.1%). Regarding *opportunity* (enablement of values), the following percentages show the teachers (from highest to lowest) who reported being enabled in the different capability components: use of knowledge and skills (81.6%), contributing to something valuable (80.1%), setting own goals (77.9%), development of knowledge and skills (77.3%), building and maintaining meaningful relationships at work (77.1%), involvement in important decisions (61.4%), and having a good income (54.3%).

**TABLE 1:** Frequencies and percentages of capabilities.

Capability	IMP	OPP	SUC	COMB
UKS	97.2 2.8	81.6 18.4	85.8 14.2	78.7 21.3
DKS	91.5 8.5	77.3 22.7	75.9 24.1	69.5 30.5
IID	82.1 17.9	61.4 38.6	59.7 40.3	54.3 45.7
MRW	91.4 8.6	77.1 22.9	82.9 17.1	75.0 25.0
SOG	90.1 9.9	77.9 22.1	80.0 20.0	73.0 27.0
CSV	92.9 7.1	80.1 19.9	78.7 21.3	73.8 26.2
HGI	88.7 11.3	54.3 45.7	58.9 41.1	50.4 49.6
EFST	95.7 4.3	81.6 18.4	87.2 12.8	76.4 23.6

*UKS, use of knowledge and skills; DKS, development of knowledge and skills; IID, involvement in important decisions; MRW, building and maintaining meaningful relationships at work; SOG, setting own goals; CSV, contributing to something valuable; HGI, having a good income; EFST, being an effective secondary school teacher; IMP, importance (value); OPP, opportunity; SUC, achievement; COMB, combined total.*

Finally, concerning *achievement*, the following percentages of teachers (from highest to lowest) reported being able to succeed in achieving each capability component: use of knowledge and skills (85.8%), building and maintaining meaningful relationships at work (82.9%), setting own goals (80%), contributing to something valuable (78.7%), development of knowledge and skills (75.9%), involvement in important decisions (59.7%), and having a good income (54.3%). For the overall question (being an effective secondary school teacher), the following percentages of teachers reported capability: 95.7% (importance of value), 81.6% (opportunity), and 87.2% (achievement).

Table 1 indicates that the percentages of secondary school teachers who reported capabilities were as follows: use of knowledge and skills (78.7%), building and maintaining meaningful relationships at work (75%), contributing to something valuable (73.8%), setting own goals (73%), development of knowledge and skills (69.5%), involvement in important decisions (54.3%), and having a good income (50.4%).

### Confirmatory Factor Analysis, Reliability, and Correlations

Confirmatory factor analysis was used to assess the fit of the measurement model of flourishing at work, organizational citizenship behavior, and intention to leave. The following fit statistics were obtained: χ^2^ = 745.65 (*df* = 418, *p* = 0.000), CFI = 0.94, TLI = 0.93, RMSEA = 0.085, SRMR = 0.08. The standardized loadings of the items on their target factors were acceptable: [emotional well-being (4 items): λ = from 0.73 to 0.86; mean = 0.80; psychological well-being (9 items): from λ = 0.66 to 0.91; mean = 0.78; social well-being (5 items): from λ = 0.79 to 0.92; mean = 0.87; organizational citizenship behavior – altruism (5 items): λ = from 0.70 to 0.83; mean = 0.77; organizational citizenship behavior – generalized compliance (5 items): λ = from 0.70 to 0.92; mean = 0.75), and intention to leave (3 items): λ = from 0.72 to 0.95; mean = 0.88], showing well-defined factors corresponding to *a priori* expectations. All latent variables were allowed to correlate (the correlations between latent variables are reported in Table 2). These values indicate an acceptable fit between the model and the observed data. Table 3 indicates that the omega coefficients of all scales were acceptable compared with the cut-off value of 0.80 (Nunnally and Bernstein, 1994).

**TABLE 2:** Correlations between the variables.

	1	2	3	4	5	6	7	8	9	10	11	12	13
(1) UKS	−	−	−	−	−	−	−	−	−	−	−	−	−
(2) DKS	0.48**^+^	−	−	−	−	−	−	−	−	−	−	−	−
(3) IID	0.35**^+^	0.51**^++^	−	−	−	−	−	−	−	−	−	−	−
(4) MRW	0.23**	0.33**^+^	0.36**	−	−	−	−	−	−	−	−	−	−
(5) SOG	0.35**^+^	0.36**^+^	0.39**	0.33**^+^	−	−	−	−	−	−	−	−	−
(6) CSV	0.24**	0.20*	0.35**	0.34**^+^	0.55**^++^	−	−	−	−	−	−	−	−
(7) HGI	0.25**	0.27**	0.20*	0.21*	0.26**	0.09	−	−	−	−	−	−	−
(8) EFST	0.42**^+^	0.33**^+^	0.36**	0.37**^+^	0.28**	0.28**	0.16	−	−	−	−	−	−
(9) EWB	0.28**	0.31**^+^	0.35**	0.32**^+^	0.34**^+^	0.23*	0.36**^+^	0.27**	−	−	−	−	−
(10) PWB	0.21*	0.27**	0.31**	0.35**^+^	0.32**^+^	0.16	0.26**	0.17	0.84**^++^	−	−	−	−
(11) SWB	0.30**^+^	0.32**^+^	0.33**	0.39**^+^	0.31**^+^	0.23*	0.20*	0.24*	0.73**^++^	0.85**^++^	−	−	−
(12) OCB-AL	0.12	0.31**^+^	0.22*	0.27**	0.38**^+^	0.19*	0.09	0.16	0.14	0.31**^+^	0.42**^+^	−	−
(13) OCB-GC	0.13	0.28**	0.24*	0.25**	0.36**^+^	0.21*	0.10	0.14	0.37**^+^	0.59**^++^	0.49**^+^	0.66**^++^	−
(14) ITL	−0.22*	−0.21*	–0.13	−0.20*	–0.18	–0.11	–0.07	–0.17	−0.49**^+^	−0.48**^+^	−0.61**^++^	0.11	–0.06

*p ≤ 0.05; **p ≤ 0.01; ^+^r > 0.30 – medium effect; ^++^r > 0.50 – large effect.

*UKS, use of knowledge and skills; DKS, development of knowledge and skills; IID, involvement in important decisions; MRW, building and maintaining meaningful relationships at work; SOG, setting own goals; CSV, contributing to something valuable; HGI, having a good income; EFST, being an effective secondary school teacher; EWB, emotional well-being; PWB, psychological well-being; SWB, social well-being; OCB-AL, organizational citizenship behavior (altruism); OCB-GC, organizational citizenship behavior (generalized compliance); ITL, intention to leave.*

**TABLE 3:** Descriptive statistics and internal consistency reliabilities of the dependent variables.

Variable	Mean	*SD*	ω
Capability set	4.76	2.05	0.77
Emotional well-being	3.57	0.95	0.85
Psychological well-being	3.73	0.84	0.91
Social well-being	3.72	1.11	0.92
OCB – altruism	3.04	1.12	0.86
OCB – generalized compliance	3.55	0.96	0.82
Intention to leave	2.69	1.26	0.87

*(a) The maximum capability set score is 7.*

*(b) Well-being and OCB means are out of a maximum of 6.*

*(c) The ITL mean is out of a maximum of 5.*

Point-biserial and Pearson correlations between the variables are reported in Table 2. Not shown in Table 2 are the correlations between the seven capabilities and the capability set for teachers: use of knowledge and skills (*r*_*s*_ = 0.57, *p* < 0.01), development of knowledge and skills (*r*_*s*_ = 0.68, *p* < 0.01), involvement in important decisions (*r*_*s*_ = 0.73, *p* < 0.01), building and maintaining meaningful relationships at work (*r*_*s*_ = 0.59, *p* < 0.01), setting own goals (*r*_*s*_ = 0.66, *p* < 0.01), contributing to something valuable (*r*_*s*_ = 0.57, *p* < 0.01), and having a good income (*r*_*s*_ = 0.57, *p* < 0.01). Thus, all seven capabilities separately had a significant correlation with the capability set as a whole, indicating that each influenced the set moderately in a positive direction.

### Multiple Regression Analyses

Multiple regression analyses were carried out with demographic variables (age, gender, school tenure, and teaching experience) and capabilities (measured by the CSWQ) as independent variables and emotional, psychological, and social well-being (measured by the FAWS–SF), organizational citizenship behavior (measured by the OCBQ), and intention to leave (measured by the TIS) as dependent variables (see Table 4).

**TABLE 4:** Multiple regression analyses of demographic variables, capabilities, and flourishing at work.

		EWB						PWB						SWB					
Capability	Variable	ß	*SE*	*p*	*R* ^2^	*F*	*p*	ß	*SE*	*P*	*R* ^2^	*F*	*p*	ß	*SE*	*p*	*R* ^2^	*F*	*p*
UKS	Age	0.15	0.09	0.36	0.14	2.55	0.04*	0.26	0.09	0.09	0.18	3.43	0.01**	0.28	0.12	0.06	0.21	4.03	0.01**
	Gender	–0.05	0.15	0.67				–0.12	0.13	0.26				–0.14	0.18	0.2			
	Tenure	–0.07	0.11	0.73				0.08	0.1	0.67				0.01	0.13	0.95			
	Experience	–0.17	0.12	0.4				–0.45	0.11	0.02*				–0.43	0.15	0.03*			
	UKS	0.32	0.21	0.00**				0.28	0.19	0.01**				0.3	0.26	0.01**			
DKS	Age	0.19	0.09	0.23	0.15	2.83	0.02*	0.29	0.08	0.05*	0.21	4.23	0.00**	0.31	0.11	0.03*	0.25	5.25	0.00**
	Gender	–0.02	0.14	0.82				–0.1	0.13	0.34				–0.11	0.18	0.26			
	Tenure	–0.1	0.11	0.57				0.05	0.1	0.79				–0.02	0.13	0.9			
	Experience	–0.18	0.12	0.35				–0.47	0.11	0.02*				–0.45	0.14	0.02*			
	DKS	0.34	0.17	0.00**				0.34	0.16	0.00**				0.36	0.22	0.00**			
IID	Age	0.17	0.09	0.27	0.17	3.11	0.01**	0.28	0.08	0.06	0.22	4.28	0.00**	0.31	0.11	0.04*	0.23	4.6	0.00**
	Gender	–0.02	0.14	0.89				–0.09	0.13	0.38				–0.11	0.18	0.31			
	Tenure	–0.19	0.11	0.3				–0.04	0.1	0.83				–0.11	0.13	0.55			
	Experience	–0.14	0.12	0.47				–0.43	0.11	0.03*				–0.41	0.15	0.03*			
	IID	0.37	0.16	0.00**				0.35	0.14	0.00**				0.34	0.2	0.00**			
MRW	Age	0.12	0.09	0.46	0.14	2.54	0.04*	0.21	0.08	0.16	0.22	4.43	0.00**	0.22	0.11	0.14	0.28	6.11	0.00**
	Gender	–0.03	0.15	0.76				–0.11	0.13	0.29				–0.13	0.17	0.21			
	Tenure	–0.18	0.11	0.34				–0.03	0.1	0.86				–0.11	0.13	0.52			
	Experience	–0.06	0.12	0.77				–0.33	0.11	0.09				–0.29	0.14	0.12			
	MRW	0.33	0.19	0.00**				0.36	0.17	0.00**				0.42	0.23	0.00**			
SOG	Age	0.17	0.09	0.27	0.16	2.89	0.02*	0.28	0.08	0.07	0.21	4.25	0.00**	0.3	0.11	0.04*	0.24	4.9	0.00**
	Gender	–0.01	0.14	0.89				–0.09	0.13	0.38				–0.1	0.18	0.31			
	Tenure	–0.14	0.11	0.46				0.02	0.1	0.93				–0.06	0.13	0.75			
	Experience	–0.14	0.12	0.49				–0.42	0.11	0.03*				–0.4	0.14	0.03*			
	SOG	0.34	0.16	0.00**				0.34	0.15	0.00**				0.35	0.21	0.00**			
HGI	Age	0.15	0.09	0.35	0.15	2.65	0.03*	0.28	0.09	0.07	0.14	2.61	0.03*	0.33	0.12	0.04*	0.14	2.48	0.04*
	Gender	–0.06	0.15	0.59				–0.12	0.14	0.26				–0.13	0.19	0.24			
	Tenure	–0.05	0.11	0.77				0.07	0.1	0.71				–0.02	0.14	0.94			
	Experience	–0.09	0.12	0.67				–0.41	0.11	0.05*				–0.41	0.16	0.05*			
	HGI	0.34	0.16	0.00**				0.21	0.15	0.06				0.14	0.21	0.23			
EFST	Age	0.16	0.1	0.33	0.09	1.61	0.17	0.28	0.09	0.08	0.13	2.42	0.04*	0.3	0.12	0.06	0.16	2.94	0.02*
	Gender	–0.01	0.15	0.95				–0.09	0.14	0.42				–0.1	0.19	0.36			
	Tenure	–0.1	0.11	0.59				0.04	0.1	0.81				–0.02	0.14	0.9			
	Experience	–0.17	0.12	0.41				–0.46	0.11	0.02*				–0.44	0.15	0.03*			
	EFST	0.24	0.21	0.04*				0.18	0.19	0.1				0.2	0.26	0.06			
Capset	Age	0.08	0.09	0.58	0.25	5.19	0.00**	0.2	0.08	0.17	0.28	6.12	0.00**	0.22	0.11	0.12	0.31	7.7	0.00**
	Gender	–0.04	0.14	0.7				–0.11	0.13	0.25				–0.13	0.17	0.19			
	Tenure	–0.11	0.1	0.52				0.04	0.09	0.82				–0.03	0.13	0.85			
	Experience	–0.1	0.11	0.6				–0.39	0.1	0.04*				–0.37	0.14	0.04*			
	Capset	0.47	0.04	0.00**				0.43	0.03	0.00**				0.45	0.04	0.00**			

*UKS, use of knowledge and skills; DKS, development of knowledge and skills; IID, involvement in important decisions; MRW, building and maintaining meaningful relationships at work; SOG, setting own goals; CSV, contributing to something valuable; HGI, having a good income; EFST, being an effective secondary school teacher; EWB, emotional well-being; PWB, psychological well-being; SWB, social well-being; OCB-AL, organizational citizenship behavior (altruism); OCB-GC, organizational citizenship behavior (generalized compliance); ITL, intention to leave; Capset, capability set.*

*p ≤ 0.05; **p ≤ 0.01.

Table 4 indicates that the capability set predicted emotional well-being statistically significantly (β = 0.47, *p* < 0.01). The following capabilities were statistically significant predictors (*p* < 0.01) of emotional well-being in the statistically significant regression models: involvement in important decisions (β = 0.37), use of knowledge and skills (β = 0.32), development of knowledge and skills (β = 0.34), building and maintaining meaningful relationships at work (β = 0.33), setting own goals (β = 0.34), and having a good income (β = 0.34). The following variables statistically significantly predicted psychological well-being: (a) less teacher experience (β = −0.45) and use of knowledge and skills (β = 0.28); (b) age (β = 0.29), less teacher experience (β = −0.47), and development of knowledge and skills (β = 0.34); (c) less teacher experience (β = −0.43) and involvement in important decisions (β = 0.35); (d) building and maintaining meaningful relationships at work (β = 0.36); (e) less teacher experience (β = −0.42) and setting own goals (β = 0.34).

Lastly, the capability set (β = 0.45, *p* < 0.01) and less experience (β = 0.37, *p* < 0.01) statistically significantly predicted social well-being (*F* = 7.70, *p* < 0.01). The following variables were statistically significant predictors of social well-being: (a) less teacher experience (β = −0.43) and use of knowledge and skills (β = 0.30); (b) age (β = 0.31), less teacher experience (β = −0.45), and development of knowledge and skills (β = 0.36); (c) age (β = 0.31), less teacher experience (β = −0.41), and involvement in important decisions (β = 0.34); (d) building and maintaining meaningful relationships at work (β = 0.42); (e) age (β = 0.30), less teacher experience (β = −0.40), and setting own goals (β = 0.35); and (f) age (β = 0.33), less teacher experience (β = −0.47), and contributing to something valuable (β = 0.21).

The capability set and specific demographic variables predicted statistically and practically significant percentages of the variance in emotional well-being (25%, large effect), psychological well-being (28%, large effect), and social well-being (31%, large effect).

Table 5 indicates that the capability set statistically significantly predicted organizational citizenship behavior – altruism (β = 0.35, *p* < 0.01). The following capabilities are statistically significant predictors (*p* < 0.01) of organizational citizenship behavior – altruism in the statistically significant regression models: development of knowledge and skills (β = 0.29), building and maintaining meaningful relationships at work (β = 0.30), and setting own goals (β = 0.38).

**TABLE 5:** Multiple regression analyses of demographic variables, capabilities, and organizational outcomes.

		OCB-AL						OCB-GC						ITL					
CAP	IV	ß	*SE*	*p*	*R* ^2^	*F*	*p*	ß	*SE*	*p*	*R* ^2^	*F*	*p*	ß	*SE*	*p*	*R* ^2^	*F*	*p*
UKS	Age	0.18	0.1	0.28	0.07	1.11	0.36	0.28	0.1	0.08	0.11	1.9	0.1	–0.31	0.09	0.05*	0.15	2.68	0.03*
	Gender	0.07	0.16	0.54				–0.11	0.16	0.32				0.07	0.14	0.53			
	Tenure	0.14	0.11	0.46				0.15	0.12	0.42				0.11	0.1	0.57			
	Experience	–0.35	0.13	0.09				–0.51	0.13	0.01**				0.31	0.11	0.12			
	UKS	0.12	0.22	0.29				0.11	0.22	0.31				–0.22	0.2	0.04*			
DKS	Age	0.17	0.1	0.27	0.13	2.4	0.05*	0.28	0.07	0.1	0.16	2.87	0.02*	–0.33	0.09	0.03*	0.18	3.47	0.01**
	Gender	0.08	0.15	0.48				–0.1	0.34	0.15				0.05	0.14	0.62			
	Tenure	0.14	0.11	0.46				0.15	0.42	0.11				0.13	0.1	0.47			
	Experience	–0.36	0.12	0.08				–0.51	0.01	0.12				0.32	0.11	0.1			
	DKS	0.29	0.18	0.01**				0.24	0.02	0.19				–0.29	0.17	0.01**			
IID	Age	0.17	0.1	0.28	0.1	1.81	0.12	0.27	0.1	0.08	0.15	2.68	0.03*	–0.35	0.09	0.03*	0.12	2.19	0.06
	Gender	0.08	0.15	0.45				–0.1	0.15	0.37				0.05	0.14	0.66			
	Tenure	0.08	0.11	0.68				0.09	0.11	0.63				0.18	0.1	0.35			
	Experience	–0.33	0.12	0.11				–0.49	0.12	0.02*				0.31	0.11	0.13			
	IID	0.24	0.17	0.04*				0.23	0.17	0.04*				–0.16	0.16	0.16			
MRW	Age	0.11	0.1	0.51	0.14	2.46	0.04*	0.22	0.1	0.16	0.16	3.03	0.02*	–0.3	0.09	0.06	0.14	2.44	0.04*
	Gender	0.07	0.15	0.52				–0.11	0.15	0.3				0.06	0.14	0.59			
	Tenure	0.07	0.11	0.7				0.09	0.11	0.62				0.18	0.1	0.34			
	Experience	–0.24	0.12	0.24				–0.41	0.13	0.04*				0.25	0.12	0.22			
	MRW	0.3	0.2	0.01**				0.27	0.2	0.02*				–0.2	0.19	0.08			
SOG	Age	0.14	0.09	0.35	0.2	3.78	0.00**	0.25	0.09	0.1	0.21	4.16	0.00**	–0.338	0.09	0.03*	0.13	2.37	0.05*
	Gender	0.09	0.14	0.4				–0.09	0.15	0.37				0.048	0.14	0.66			
	Tenure	0.11	0.11	0.55				0.12	0.11	0.5				0.152	0.1	0.41			
	Experience	–0.31	0.12	0.12				–0.47	0.12	0.02*				0.3	0.11	0.14			
	SOG	0.38	0.16	0.00**				0.34	0.17	0.00**				–0.18	0.16	0.1			
CSV	Age	0.17	0.1	0.27	0.11	1.89	0.11	0.27	0.1	0.08	0.17	3.2	0.01**	–0.36	0.09	0.02*	0.1	1.8	0.12
	Gender	0.07	0.15	0.51				–0.11	0.15	0.32				0.05	0.14	0.63			
	Tenure	0.14	0.11	0.45				0.16	0.11	0.39				0.14	0.11	0.46			
	Experience	–0.38	0.12	0.06				–0.54	0.12	0.01**				0.33	0.12	0.11			
	CSV	0.24	0.19	0.03*				0.28	0.19	0.01**				–0.06	0.18	0.6			
HGI	Age	0.2	0.1	0.23	0.06	0.91	0.48	0.3	0.1	0.06	0.1	1.72	0.14	–0.37	0.09	0.02*	0.1	1.74	0.14
	Gender	0.07	0.16	0.53				–0.11	0.16	0.33				0.05	0.15	0.63			
	Tenure	0.13	0.12	0.49				0.14	0.12	0.45				0.14	0.11	0.45			
	Experience	–0.34	0.13	0.11				–0.5	0.13	0.02*				0.32	0.12	0.12			
	HGI	0.06	0.17	0.62				0.05	0.17	0.65				–0.01	0.16	0.94			
EFST	Age	0.16	0.1	0.32	0.07	1.25	0.3	0.28	0.1	0.09	0.11	1.9	0.1	–0.32	0.09	0.05*	0.13	2.26	0.06
	Gender	0.09	0.16	0.43				–0.1	0.16	0.39				0.04	0.14	0.71			
	Tenure	0.14	0.11	0.48				0.14	0.12	0.45				0.13	0.1	0.48			
	Experience	–0.35	0.12	0.1				–0.51	0.13	0.01**				0.32	0.11	0.12			
	EFST	0.15	0.22	0.18				0.11	0.22	0.32				–0.17	0.2	0.13			
Capset	Age	0.1	0.09	0.52	0.17	10.71	0.01**	0.21	0.1	0.17	0.2	3.83	0.00**	–0.3	0.09	0.06	0.15	2.77	0.02*
	Gender	0.07	0.15	0.53				–0.11	0.15	0.28				0.06	0.14	0.58			
	Tenure	0.13	0.11	0.47				0.14	0.11	0.43				0.14	0.1	0.45			
	Experience	–0.29	0.12	0.14				–0.45	0.12	0.02*				0.28	0.11	0.16			
	Capset	0.35	0.04	0.00**				0.33	0.04	0.00**				–0.23	0.04	0.03**			

*UKS, use of knowledge and skills; DKS, development of knowledge and skills; IID, involvement in important decisions; MRW, building and maintaining meaningful relationships at work; SOG, setting own goals; CSV, contributing to something valuable; HGI, having a good income; EFST, being an effective secondary school teacher; EWB, emotional well-being; PWB, psychological well-being; SWB, social well-being; OCB-AL, organizational citizenship behavior (altruism); OCB-GC, organizational citizenship behavior (generalized compliance); ITL, intention to leave; Capset = capability set.*

*p ≤ 0.05; **p ≤ 0.01.

Table 5 indicates that the capability set (β = 0.33, *p* < 0.01) and less experience (β = −0.45, *p* < 0.01) predicted organizational citizenship behavior – generalized compliance statistically significantly. The following capabilities were statistically significant predictors (*p* < 0.01) of organizational citizenship behavior – generalized compliance in the statistically significant regression models: (a) less experience (β = −0.49) and involvement in important decisions (β = 0.23); (b) less experience (β = −0.41) and building and maintaining meaningful relationships at work (β = 0.27); (c) less experience (β = −0.47) and setting own goals (β = 0.34); and (d) less experience (β = −0.54) and contributing to something valuable (β = 0.28).

Finally, Table 5 indicates that the capability set (β = −0.23, *p* < 0.03) predicted intention to leave statistically significantly. The following capabilities were statistically significant predictors (*p* < 0.01) of intention to leave in the statistically significant regression models: (a) lack of capability to use knowledge and skills (β = −0.22) and higher age (β = 0.31); and (b) lack of capability to develop new knowledge and skills (β = −0.29) and higher age (β = 0.32).

The capability set and specific demographic variables predicted statistically and practically significant percentages of the variance in organizational citizenship behavior – altruism (17%, medium effect), organizational citizenship behavior – generalized compliance (20%, medium effect), and intention to leave (15%, medium effect).

### Validation of Hypotheses

Concerning flourishing at work (in hypothesis 1), H1a is accepted for the associations between the following capabilities and emotional well-being: use of knowledge and skills, development of knowledge and skills, involvement in important decisions, building and maintaining meaningful relationships at work, setting own goals, and having a good income. H1b is accepted for the associations between the following capabilities and psychological well-being: use of knowledge and skills, development of knowledge and skills, involvement in important decisions, building and maintaining meaningful relationships at work, and setting own goals. H1c is accepted for the following capabilities and social well-being: knowledge and skills use, knowledge and skills development, involvement in important decisions, building and maintaining meaningful relationships at work, setting own goals, and contributing to something valuable. Regarding organizational citizenship behavior, H1d is accepted for the following capabilities and altruism: development of knowledge and skills, building and maintaining meaningful relationships at work, and setting goals. H1e is accepted for the following capabilities and generalized compliance: involvement in important decisions, building and maintaining meaningful relationships at work, setting own goals, and contributing to something valuable. Finally, regarding intention to leave, H1f is accepted for the lack of the following capabilities: use knowledge and skills and develop new knowledge and skills.

Concerning hypothesis 2, H2a is accepted. All capabilities were statistically significantly and strongly related to the capability set. Finally, regarding hypothesis 3, H3a–H3f are accepted, suggesting that the capability set of secondary school teachers was significantly associated with all three flourishing dimensions, two organizational citizenship dimensions, and intention to leave.

## Discussion

This study aimed to identify work capabilities of secondary school teachers – valued aspects of work that are enabled and can be realized – and investigate their effects on three functionings: flourishing at work, intention to leave, and organizational citizenship behavior. The results showed that 82.1% to 97.2% of the teachers regarded each of the seven values as important. Most teachers valued using knowledge and skills and contributing to something valuable. The least number of teachers valued involvement in decision-making. The enablement responses indicated that 54.3–81.6% of secondary school teachers perceived themselves as enabled in each capability. Most of the teachers indicated enablement concerning knowledge and skills. However, the lowest enablement was reported for having a good income. From 58.9 to 85.8% of the teachers reported that they had successfully achieved the seven capabilities. The use of knowledge and skills was a capability for most teachers, while having a good income was the least achieved capability. When the three elements (value, opportunity, and achievement) were combined in capabilities, 78.7% of respondents reported overall capability in the use of knowledge and skills (most reported) and 50.4% in having a good income (least reported).

Most teachers reported all seven work values as being important to them. However, they most valued using and developing knowledge and skills, contributing to something valuable, building and maintaining meaningful relationships at work, and setting their own goals. Moreover, having a good income and involvement in important decisions were also important to more than 80% of the teachers. Enablement and opportunities to realize their values were considerably lower for all values, but even more so for having a good income and involvement in important decisions. Achievement of capabilities showed the same pattern as enablement: achievement was considerably lower than importance for all seven values, but specifically for having a good income and involvement in important decisions. These findings are in line with findings from other studies (e.g., Abma et al., 2016; Van Gorp et al., 2018), which postulated that workers with differences between enablement and achievement of capabilities might be influenced by something outside the work situation that may be keeping them from achieving the work capability.

The participants perceived their capabilities as average to high in using their knowledge and skills, building and maintaining meaningful relationships at work, contributing to something valuable, and setting their own goals. Less capability was indicated regarding developing their knowledge and skills, being involved in important decisions, and having a good income. Overall, the value attached to capabilities was generally higher than the opportunity for and achievement of these capabilities. These results concur with findings in previous studies (Abma et al., 2016), although secondary school teachers indicated more success than enablement in four of the capabilities (use of knowledge and skills, building and maintaining meaningful relationships at work, setting own goals, and having a good income).

The capability set and specific demographic variables predicted significant and large percentages of the emotional, psychological, and social well-being variance. This is in line with findings of other studies that capabilities relate to better work outcomes (Van Gorp et al., 2018; Van Casteren et al., 2021). Demographic variables and the capability set significantly and positively predicted emotional, psychological, and social well-being and organizational citizenship behavior (altruism and generalized compliance), and negatively predicted intention to leave. The findings revealed that the different capabilities and demographic variables (specifically age and experience) predicted the dependent variables in various permutations. Gender and tenure had no practically significant effect on the flourishing variables.

Each capability predicted emotional well-being positively, except for contributing to something valuable. Therefore, knowledge and skills use, development of knowledge and skills, involvement in important decisions, building and maintaining meaningful relationships at work, setting own goals, and having a good income are associated with emotional well-being (i.e., positive affect and job satisfaction). However, the capability set of secondary school teachers (which combines the seven values, their enablement, and achievement) was a better predictor of emotional well-being than a specific capability. Emotional well-being (i.e., feeling well) results from individuals’ *needs* satisfaction and realizing their *wants* (Rojas and Veenhoven, 2013). The satisfaction component of emotional well-being relates to teachers’ perceptions of all aspects of their current jobs in terms of what they think can be, while positive affect is linked to the gratification of their needs.

Each capability, except having a good income and contributing to something valuable, positively predicted psychological well-being. More specifically, knowledge and skills use, knowledge and skills development, involvement in important decisions, building and maintaining meaningful relationships at work, and setting own goals were associated with functioning psychologically well. However, the capability set was a better predictor of psychological well-being than a specific capability. Therefore, capabilities were associated with psychological need satisfaction (i.e., autonomy, competence, and relatedness, Deci and Ryan, 2011), learning (Spreitzer et al., 2012), meaningful work (Steger et al., 2012), and engagement (Kahn and Heaphy, 2014).

Different capabilities, except having a good income, positively predicted social well-being. Specific capabilities, including knowledge and skills use, knowledge and skills development, involvement in important decisions, building and maintaining meaningful relationships at work, setting own goals, and contributing to something valuable, were associated with functioning socially well. However, the capability set was a better predictor of social well-being than a specific capability. Therefore, teacher capabilities are associated with feeling less isolated and more connected and believing they matter (Son and Wilson, 2012; Prillentensky and Prillentensky, 2021).

Specific capabilities and the capability set were associated with the altruism dimension of organizational citizenship behavior. Capabilities associated with altruism (as a dimension of organizational citizenship behavior) included developing knowledge and skills, building and maintaining meaningful relationships, and setting goals. Use of knowledge and skills, having a good income, and the various demographic variables did not significantly affect altruism. Generalized compliance (a dimension of organizational citizenship behavior) was positively associated with less teaching experience combined with the capabilities to be involved in important decisions, build and maintain meaningful relationships at work, set own goals, and contribute to something valuable. Neither use and development of knowledge and skills nor having a good income significantly affected generalized compliance. Both dimensions of organizational citizenship behavior were associated with the capability set of teachers. Building on the findings of Möller and Rothmann (2019), organizational citizenship behavior as a type of performance is linked to work capabilities.

Lastly, the intention to leave was associated with younger age, and lack of two capabilities, namely *using* and *developing* knowledge and skills. The capabilities of being involved in important decisions, setting own goals, contributing to something valuable, and having a good income did not significantly affect secondary school teachers’ intentions to leave. Whilst various studies (e.g., Jackson et al., 2006; Fouché et al., 2017) have linked intentions to leave to a lack of job resources, this study suggests that teacher capabilities matter. In this regard, resources that affect knowledge and skills use and development probably matter most.

The results showed that the larger the capability set was, the higher the scores for emotional, psychological, and social well-being and organizational citizenship behavior. Furthermore, the lower the capability set was, the more teachers indicated that they wanted to leave their jobs. Thus, if teachers managed to achieve what they valued in work, they reported better emotional, psychological, and social well-being. They were more inclined to show organizational citizenship behavior (i.e., altruism and generalized compliance) and were less inclined to resign. The results also revealed that some capabilities seemed to have a minimal effect on predicting flourishing at work. For example, contributing to something valuable had no significant effect on increasing employee well-being or lowering intention to leave; having a good income had no significant effect on increasing psychological or social well-being, as well as organizational citizenship behavior, or lowering intention to leave.

The use of knowledge and skills seemed to have no significant effect on organizational citizenship behavior. The capability to be involved in important decisions did not affect intention to leave. This is in line with other studies in developing countries that showed that payment of an allowance to rural teachers had no significant effect on teacher characteristics or learner test results (Chelwa et al., 2019). In another country, learner performance, on average, was not affected by financial incentives, but worked out better for high-performing learners and worse for low-performing for rural primary school learners (Pugatch and Schroeder, 2014). However, the results of this study indicated that the capability set had a meaningful impact on all the measured flourishing variables, which was aligned with the motivational-hygiene model (Herzberg et al., 2010). This model states that employee motivation is achieved when balance occurs between the challenge and the pleasure of work – where employees are enabled to do and be a variety of aspects that they value, such as achieving, growing, demonstrating responsibility, and progressing in the organization.

Less teaching experience combined with knowledge and skills use, knowledge and skills development, involvement in important decisions, and setting own goals to affect psychological well-being. Notably, the capability set combined with less teaching experience were associated with psychological and social well-being. A reason for the effects of fewer years of teaching experience is that teachers with less experience completed their teacher education more recently, which might have contributed to their capability to achieve well-being. Interestingly, age (being older) and less experience contributed positively and significantly to the capabilities to be involved in important decisions, set own goals, and contribute to something valuable. Less experience significantly affected psychological well-being, social well-being, and organizational citizenship behavior (generalized compliance). However, the capability set and less teaching experience positively and significantly predicted generalized compliance.

Due to every aspect of educational transformation depending on competent teachers for its achievement (Pretorius, 2013; Eyre, 2016), a stronger focus on teachers and school leaders as drivers for change is pertinent. Therefore, shaping the teaching profession is becoming core to policymakers and schools, bringing about high-performance learning cultures for teachers to develop learners ready for life after school. However, research reports that there are differences between how teachers and policymakers interpret teachers’ work and what is valued and considered good-quality teaching from these two perspectives (Buckler, 2012). The findings in this study provide important insights into understanding secondary school teacher functionality in South Africa by indicating that giving teachers what they need may lead to better outcomes for the organization. It also provides insight into the specificity of the functionings that are impacted by the different secondary school teachers’ capabilities, which could assist in focusing interventions in specific ways to ensure optimized outcomes for the organization.

## Limitations and Recommendations for Future Research

This study had various limitations. Firstly, a cross-sectional survey was used. Longitudinal data are needed to measure the impact of capabilities on well-being over time (Fleuren et al., 2016). Secondly, the sample was relatively small, cautioning against generalizing findings before further data had validated these. Moreover, the timing of the data collection phase (at the beginning of the COVID-19 lockdown period in South Africa) could have played a role in participant sentiment, further making a case for a longitudinal study of the topic. The voluntary nature of study participation and the demands on teachers because of the COVID-19 pandemic made it very difficult to source participants. Additionally, the sample was small because principals and teachers were under pressure to cover the curriculum in a short time when data collection took place. Furthermore, the social distancing protocols prescribed during the pandemic lockdown made it challenging to obtain more participants. Thirdly, a capability framework was used in a quantitative survey design (Anand and Van Hees, 2006). Although this was offset by utilizing an existing framework (Van der Klink et al., 2016), as well as data that included subjective experiences through the reporting of value, opportunity, and achievement, it would be prudent to validate the study results from a cultural perspective using qualitative research design.

Three capabilities seemed lacking in this sample of secondary school teachers in the Tshwane districts: developing new knowledge and skills, involvement in important decisions, and having a good income. These are important targets for interventions by schools and the education department. It would be beneficial for educational leaders to apply an autonomy-supportive leadership style (Rothmann and Fouché, 2018). Other studies (e.g., Venter and Viljoen, 2020) indicated that growing the number of teachers in educational institutions might have a more positive impact on a school system than growing the quality of the existing teachers, but aggregating both quantity and quality would be the best tactic.

Future research on the capabilities of secondary school teachers should investigate the effects of capability enablement in different schooling contexts. A better understanding of the effects of teaching experience on capabilities and functionings is also needed. Moreover, further research can investigate the effect of capabilities on teachers’ perceptions of their job performance.

## Conclusion

This study determined the status of sustainable employability capabilities of a sample of secondary school teachers in the Tshwane districts of Gauteng, South Africa. These were measured according to the value, enablement, and achievement teachers perceived in each aspect and overall capability. The effects of these capabilities on specific work functionings were provided as reported by the teachers. The results indicated that the capability set had the best predictive value for flourishing at work functionings. They also showed that teaching experience (mostly) and age (to a lesser extent) played a role in predicting flourishing at work in combination with specific capabilities. Overall, the results indicated that sustainable employability capabilities were a factor in facilitating teachers’ functionings. However, further research is needed to ascertain how changes in capability affect the teacher and learner performance alike, specifically in different teaching contexts. In addition, more research is needed to understand why less teaching experience increases sustainable employability capability.

## Data Availability Statement

The raw data supporting the conclusions of this article will be made available by the authors, without undue reservation.

## Ethics Statement

The studies involving human participants were reviewed and approved by Health Research Ethics Committee (HREC) at North-West University (NWU-00430-19-A1). The patients/participants provided their written informed consent to participate in this study.

## Author Contributions

TDW took the lead in conceptualizing, wrote the manuscript, and collected and analyzed the data. SR acted as additional writer and reviewed the manuscript. Both authors contributed to the article and approved the submitted version.

## Conflict of Interest

The authors declare that the research was conducted in the absence of any commercial or financial relationships that could be construed as a potential conflict of interest.

## Publisher’s Note

All claims expressed in this article are solely those of the authors and do not necessarily represent those of their affiliated organizations, or those of the publisher, the editors and the reviewers. Any product that may be evaluated in this article, or claim that may be made by its manufacturer, is not guaranteed or endorsed by the publisher.
